# An Estimate of the Excess of the Heat and Cold of the American Atmosphere beyond the European, in the Same Parallel of Latitude: To Which Are Added, Some Thoughts on the Causes of This Excess

**Published:** 1797

**Authors:** Edward Augustus Holyoke


### XIX. *An Eſtimate of the Exceſs of the Heat and Cold of the American Atmoſphere beyond the European, in the ſame Parallel of Latitude: to which are added, ſome Thoughts on the Cauſes of this Exceſs.* *By* Edward Aug. Holyoke, *M. D. F. A. A.*—From the *Memoirs of the American Academy of Arts and Sciences*, Vol. II. Part I. 4to. Boſton, 1793.

### PART I.

ALMOST from the firſt diſcovery of North America, it has been obſerved, that the extremes of heat and cold are much greater on this ſide the Atlantic ocean, than they are in Europe, under the ſame parallel of latitude. But the quantity of this difference has not hitherto, ſo far as I am acquainted, been an object of much attention, or been determined with any degree of exactneſs. A valuable work, publiſhed, for ſome years paſt, by a Meteorological Society at Manheim, in Germany, entitled *Ephemerides Meteorologicæ Pala-*

*tinæ*, affords data for determining this point more precifely, as it contains more numerous and more accurate obfervations than any other publication extant.

I have therefore, from this collection, formed a table of the greateft heat and greateft cold, and of the mean of the greateft heat and cold, for a courfe of years, of twenty different cities in Europe; the fouthernmoft of which is Rome, in lat. 41° 53′, a few minutes fouthward of Bofton; and the northernmoft, Stockholm, the capital of Sweden, in lat. 59° 20′, comprehending an extent of upwards of 17° of latitude; and from Rochelle, on the weftern coaft of France, to Buda, the capital of Hungary, comprehending 20° of longitude; which takes in all the middle region of Europe. To which are added, my own obfervations of the greateft heat and cold, &c. made at Salem, in Maffachufetts.

By this table it appears, that of the twenty European cities mentioned in it, the thermometer was higheft at Wartzburgh, in the circle of Franconia, viz. 102°. 4, which falls fhort of our greateft heat above 3 degrees. The greateft degree of cold happened at Sagan, a city in the weftern borders of Silefia. There the the mercury in the thermometer funk to—21°. 32, which exceeds our greateft cold at Salem by 10°. 3; but is juft as low, as we were informed by the public prints at the time, though I know not upon what authority, that the thermometer fell at Hartford, in Connecticut, and at New York, in the month of January, 1786. But what is moft to our purpofe, the *mean* of the greateft heat in all thofe places, taken collectively, for the period noted in the third column of the table, amounted to no more than + 86°. 41, which is more than 10° fhort of our greateft heat at Salem: and the mean of the greateft cold in thefe twenty cities, amounted to + 3°. 31, which is fhort of the mean of our greateft cold upwards of 5 degrees.

But in order to determine the difference between our heat and cold, and the European, in the fame latitude, we muft compare with thofe cities, which are fituated in latitudes neareft our own, viz. Padua, Marfeilles, and Rome. We find by the table, that the mean of their greateft heat falls fhort of ours 5°. 62, 7°. 42, and 11°. 59, refpectively. We alfo find the mean of the greateft cold of thefe three cities is lefs than ours by 19°. 41, 29°. 92, and 35°. 88, refpectively. Further, the mean of the greateſt heat of theſe three cities, taken collectively, which is 88°. 1, deducted from the mean of our greateſt heat, which is 97°. 02, leaves a difference of 8°. 92 hotter. And the mean of the greateſt cold of theſe cities, being + 25°. 96, taken from the mean of our greateſt cold, —2°. 42, gives a difference of 28°. 38 colder.

The air of America then, in our latitude, is hotter in ſummer (when hotteſt) by 10 degrees of Fahrenheit's thermometer*, and colder in winter (when coldeſt) by 5 degrees, than the whole middle region of Europe taken collectively, whoſe mean latitude is about 49° or 50°, that is, about 7 or 8 degrees more northerly than Boſton.

Again, the air in America is hotter in ſummer, by upwards of 8 degrees, and colder in winter, by 28 degrees, than thoſe parts of Europe, which lie nearly in the ſame latitude †.

* I have all along made uſe of Fahrenheit's ſcale, as being much more familiar to us than Reaumur's.

† As theſe three cities, taken together, lie a degree or two northward of us, the reſult here given is rather leſs, than the true.

### PART II.

Here then is a very notable difference in reſpect both of heat and cold, in two tracts of our globe, which equally enjoy the influence of the ſun, that prime ſource of heat to our ſyſtem; and it is much greater, I believe, than any one would imagine, who had not attended to obſervations of this kind: which naturally leads to an inquiry into the cauſe of ſo remarkable an exceſs; for this cauſe ſtill remains a problem, which has never, I ſuppoſe, received a ſatisfactory ſolution.

Various conjectures have been formed upon the ſubject; one or two of which I will mention. The firſt, and, perhaps, the moſt commonly received opinion among us, is, "That "thoſe lakes and large tracts of inland waters, "which lie back of our ſettlements, being "conſtantly frozen over every winter, expoſe "a large ſurface of ice to the air; which be-"ing hereby rendered very cold, and being "ſoon wafted to the ſea-coaſt, where our moſt "numerous ſettlements are ſituated, occaſions "that degree of cold, which the inhabitants

"of North America ſuffer beyond the Europeans "in the ſame climate." To which I object, 1ſt. That the winds, which for the moſt part produce our moſt intenſe cold, are not weſterly, as upon this hypotheſis they ought to be, but north weſterly, or ſtill more northerly winds, which laſt certainly do not blow over any great extent of water in their paſſage to our coaſts. 2d, The cauſe aſſigned does not ſeem adequate to the effect; for though the lakes to the weſtward of us are large, yet they bear but a ſmall proportion to the extent of land over which the winds muſt paſs, ere they arrive at our ſettlements. 3d, I ſuppoſe that a ſurface of frozen water does not render the air, that paſſes over it, in any degree colder, than an equal ſurface of frozen earth; for frozen earth is as cold as frozen water; and all the ſurface of the ground, between the lakes and us, is frozen every winter, before the lakes themſelves are. And, if they do not render the air colder, after they are frozen, than an equal ſurface of frozen earth; certainly it cannot be ſuppoſed that they increaſe the cold, before that period. Add to this, 4th, That this ſurface of ice, which covers the lakes every winter, is pretty early in the ſeaſon clothed with ſnow, more or leſs deep, as well as the whole ſurface of theſe northern countries: now, no one can ſuppoſe that ſnow, becauſe it lies upon a ſurface of water or ice, is capable of producing a greater degree of cold in the atmoſphere, than if it covered an equal extent of ground. Theſe obſervations do, I think, evidently ſhow, that this hypotheſis is not admiſſible.

Others have ſuppoſed, "that our woods and " thick foreſts, by harbouring large quanti- " ties of ſnow every winter, and ſcreening it " from the action of the ſun's rays, do occa- " ſion the air, which blows over it, to be much " colder than it would be otherwiſe, or than " it is in Europe." This may, indeed, in ſome meaſure, account for the length of our winters, and the ſharpneſs of the winds in the ſpring; as the ſnow will remain longer undiſſolved, when ſhaded from the ſun, than in the open and cultivated parts of the country: but I do not conceive how the ſnow, which lies in the woods, ſhould communicate any extraordinary keenneſs to the air, beyond that which covers the ground every where in theſe regions, whether cleared or uncleared, during the winter ſeaſon.

However, that the woods of America are

ſomehow the occaſion of its greater cold, muſt, I believe, be admitted.

Several writers have obſerved, that the winter's cold in the old continent was formerly much more ſevere and intenſe in the ſame climate and the ſame ſpot, than it is at this day. This is a remarkable fact, of which, however, I imagine there is ſufficient proof. For ſevere froſts are mentioned by ancient authors as common events, in particular places, where nothing of the kind occurs now; or, if at ſome diſtant intervals they do ſtill ſometimes happen, they are conſtantly noted as very extraordinary.

David, King of Iſrael, in one of his pſalms*, ſays, "He giveth ſnow like wool, he ſcattereth "the hoar froſt like aſhes. He caſteth forth "his ice like morſels; who can ſtand before "his cold?" And about the time of our Saviour's crucifixion (about the beginning of April) St. John tells us†, that "the ſervants "and officers had made a fire of coals (for it was cold) and they warmed themſelves."

And Juvenal makes mention of the freezing of the river Tiber, as a common event in his

* Pſalm cxlvii. v. 16, 17. It is not material whether David was the author of this pſalm, or not.

† John, ch. xviii. v. 18.

time time*. And Ovid talks of frozen wine in countries, where, I ſuppoſe, very ſevere froſts are now very unuſual †.

If any doubt reſpecting this point ſhould ſtill remain, I believe it will be much leſſened by an attention to what Livy, the Roman hiſtorian, relates, in his account of the ſecond Punic war. There we find, that when the Romans, under the command of Scipio, beſieged a town in Spain, near the river Ebro, in a latitude a little more ſouthward than ours, he ſays, "*Nec obſeſſos alia ulla res, quàm iniqua oppug-" "nantibus hyems tutabatur. Triginta dies obſi-" "dio fuit; per quos raro unquam nix minus qua-" "tuor pedes alta jacuit: adeoque pluteos ac vi-" "neas Romanorum operuerat, ut ea ſola, ignibus" "aliquoties conjectis ab hoſte, etiam tutamentum" "fuerit.*" (Lib. xxi.) That ſnow ſhould lie four feet deep on the ground for thirty days together at Taragona or Barcelona, (in the neighbourhood of which this town lay) would, at the preſent day, be looked on as a moſt extraordi-

* Hybernum fracta glacie deſcendit in amnem, &c. Sat. vi. l. 521.

† Nudaque conſiſtunt formam ſervantia teſtæ

Vina: nec hauſta meri, ſed data fruſta bibunt. *Triſt. Lib.* 3. *Eleg.* x.

nary nary phenomenon indeed. See alſo Virgil's 3d Georgic.

And, as no change has taken place upon the ſurface of the earth in that continent, that we are acquainted with, ſo remarkable, and ſo likely to have any great influence upon the atmoſphere, as that of cutting down and clearing the earth's ſurface of thoſe woods and thick foreſts that abounded every where; may we not probably conjecture that this circumſtance is ſomehow the cauſe, why it is warmer in Paleſtine now, than in the days of King David; and at Rome, than it was in the times of the commonwealth, or of the Cæſars *?

Now, it appears highly probable, that the ſame cauſe, whatever it was, which rendered Europe colder formerly than at preſent, makes America at this day colder than Europe. America is at this day in a ſituation ſimilar to that

* It appears by the annexed table, that at Rome there does not happen every year, at this day, ſuch a degree of cold, as to ſink the thermometer down to the freezing point. The difference then between its atmoſphere in Juvenal's days, and the preſent, muſt be very great. And, as to Jeruſalem, as it lies about ten degrees farther ſouth than Rome, I preſume that nothing like a froſt ever happens there, at this day.

which Europe was in, with reſpect to its woods, thirty, or perhaps thirty-five, centuries ago. Its ſurface, excepting about a hundred or a hundred and fifty miles, more or leſs, along the ſea coaſt, is almoſt univerſally covered with thick and almoſt impenetrable foreſts, as is well known to every one. And, as the ſame cauſes always produce the ſame effects, it ſeems very probable, that the foreſts of America are, in ſome way or other, inſtrumental in producing that extra degree of cold, for which our winters are ſo remarkable.

Taking this, therefore, for a probable ſuppoſition, let us purſue it, and inquire whether it be confirmed by reaſon and experiment.

Among the many happy diſcoveries in philoſophy and chemiſtry, with which the celebrated Dr. Prieſtley has obliged the world, one of very great importance is, "The property, "which the leaves of all plants and vegeta-"bles of every kind poſſeſs, of yielding, in "day-light, air of a much purer kind, freer "from phlogiſton, and fitter for reſpiration, "than common atmoſpheric air: that they not "only furniſh large quantities of ſuch air, but "have alſo the faculty of abſorbing phlogiſton "from air, when fouled by a mixture of it,

"ſo

" ſo as to render the ſame ſalubrious and reſpi-

" rable, which was before noxious and ſuffo-

" cating; and thus become, in the hands of

" the great Author of Nature, one grand cor-

" rector of thoſe impurities which might other-

" wiſe ſo far increaſe, as to contaminate the

" whole maſs of the atmoſphere; and in pro-

" ceſs of time render it totally unfit for reſpi-

" ration, and the ſupport of animal life."

This is a doctrine well eſtabliſhed, and needs no new proofs.

All vegetables, then, both in Europe and America, are continually ſupplying the atmoſphere with this pure air, and counteracting thoſe phlogiſticating proceſſes, ſuch as combuſtion, reſpiration, putrefaction, &c. which are continually going on in all parts. But there is this material difference between the two continents: in Europe, upon the coming on of the froſty ſeaſon, the leaves of all vegetables, on or near the earth's ſurface, languiſh; and, if they do not die, yet moſt probably they perform their office of dephlogiſticating the air, in a much more languid manner, than in ſummer; or are perhaps entirely covered with ſnow, which, while it continues on them, muſt effectually put a ſtop to this proceſs; and as to the trees, their leaves for the moſt part drop off, and no more pure air is to be expected from them, till they are again renewed in the ſpring. But in America, although the leaves of all vegetables on the earth's ſurface are frozen and killed early in the winter, and the leaves of many of our trees fall off, and yield no more of this pure air than the European trees, yet there is a conſtant and large ſupply of it, from thoſe vaſt quantities of pine trees, firs, ſpruce, cedars, junipers, ſavins, hemlocks, and other ever-greens, which retain their leaves through the intenſeſt froſts, and which do greatly abound in our American woods, from the 30th to the 50th degree of N. latitude; a quantity ſufficient, perhaps, to cover one 5th or 6th of the whole ſurface of the continent of Europe.

That our evergreens do in fact yield, during the winter ſeaſon, ſuch a pure air, I have ſeveral times found by experiment. My experiments were conducted in the manner in which Drs. Prieſtley and Ingenhouſz conducted theirs; and I conſtantly found air produced from the leaves of juniper and pine (the only ones I have yet made trial of) in the ſame manner as from from other leaves in ſummer *. But it ought to be obſerved, that this manner of experimenting cannot be proſecuted, when the water is colder than 32 degrees by Fahrenheit's thermometer, as the water would then be converted into ice; but it appears reaſonable to ſuppoſe, if as much air is produced in theſe experiments, when the thermometer is at 34° or 35°, as when it mounts to 70° or 80°, that the weather, though much colder, would make no material alteration in the reſult. This fact then is not to be conſidered as fully eſtabliſhed (though, I believe, whenever proper and deciſive experiments are made, it will be confirmed) but as in a good degree probable.

This being allowed, what a fund of pure de-

* I have not yet had either time or opportunity to proſecute experiments upon evergreens as I could wiſh, or as I promiſe myſelf I may have; but, from what I have experienced, I think it no extravagant ſuppoſition, that a pine tree, of a common ſize, ſhould yield four barrels of dephlogiſticated air in one clear fair day; to which, if we add, that as much phlogiſton is abſorbed in the ſame time as an equal quantity of atmoſpheric air contains, we may readily imagine, that, in a country abounding with trees of this kind, the atmoſphere muſt be much more dephlogiſticated in winter, than in a country where evergreens are rarely met with.

phlogiſtiphlogiſticated air have we here in America, beyond what Europe at preſent poſſeſſes. There are no doubt evergreens of various kinds ſcattered all over Europe; yet it cannot be ſuppoſed, that the quantities bear any proportion to thoſe which once flouriſhed there; eſpecially in the ſouthern and middle regions of that continent; and as to Scandinavia, where I ſuppoſe they abound moſt at preſent, I imagine they muſt be very much thinned by this time. But what will all theſe amount to, when brought into a compariſon with the evergreens of America?

From theſe conſiderations, I think it muſt appear highly probable that America is furniſhed with ſources of dephlogiſticated air, which are now exhauſted in Europe; and that therefore, moſt probably, its atmoſphere is really more pure and dephlogiſticated.

Whether this be in reality the fact, may be determined moſt ſatisfactorily from meteorological obſervations. For the ſenſible qualities of the atmoſphere, which are the objects of meteorology, may, if properly attended to, and noted down for a courſe of years, determine not only which country enjoys the dryeſt and pureſt air, but alſo the quantity of the difference difference (if any there be) may as eaſily and as preciſely be known, as the difference of their heat and cold.

To ſuch obſervations then we muſt recur; and if we are enabled to determine the quantity of evaporation, the quantity of rain, the number of clear fair days, the number of cloudy days, of rainy days, and of foggy days, in each continent, for a competent courſe of years, there is no doubt but the point may be ſatisfactorily determined. This I ſhall next attempt; after premiſing that we have not yet, perhaps, obſervations enough to ſettle the matter without all doubt, yet enough, I imagine, to ſhow that it is in the higheſt degree probable, that the climate of America is much dryer in general, than that of Europe*.

The quantity of evaporation in any country muſt, I think, depend principally, if not entirely, upon the three circumſtances of dryneſs, heat, and motion of the air, contiguous to the

* Perhaps the moſt direct way of determining the dryneſs of the atmoſphere is by the hygrometer; but till this inſtrument is more improved than at preſent, and obſervations have been made upon it for ſome competent time, in both continents, this mode of determining it muſt remain a deſideratum.

evaporating ſurface*. For the dryer the air, the more capable it is of abſorbing a certain quantity of water in a given time; for when fully ſaturated with water, as in a foggy ſeaſon, little or no evaporation takes place. Heat too is found to promote evaporation, probably as it leſſens the coheſion of the particles of water: and the wind, not only by agitating the evaporating ſurface, but alſo by applying freſh portions of air to the ſame, tends greatly to promote this proceſs.

Now all theſe circumſtances conſpire with us in America, in a greater degree than in Europe, to increaſe the dryneſs upon the ſurface of the earth. And ſuch a degree of dryneſs does in fact take place here, as much more frequently to injure our crops, and fruſtrate the hopes of the huſbandman, than in Europe.

The proof of this point, however, from actual obſervation, according to the *Ephemerides Meteorologicæ*, is rather lame; for of the ſix or ſeven places mentioned in that work, which can be eaſily brought into a compariſon with

* Electricity may perhaps be conſidered as another cauſe promotive of evaporation; but then I ſuppoſe it probable, that its effect in promoting evaporation may be very much in proportion to the dryneſs of the atmoſphere.

thofe of Dr. Williams in the fame work, which are the only American ones that I have met with, two, if I underftand them, exceed his confiderably: all the reft indeed fall much fhort; for the mean evaporation of thofe feven places does not amount to quite 45 inches for the year 1785; whereas the evaporation at Cambridge the fame year, by Dr. Williams's account, was upwards of 56 inches.

The great difference in the quantity of rain which falls, in different countries, annually, makes this a remarkable article in the meteorological regifter. We are informed by Dr. Lind *, that at Senegal, in Africa, there falls, in the four rainy months, 115 inches depth of rain; and by the *Ephemerides* fo often quoted, that at St. Peterfburgh, in Ruffia, in the year 1785, there fell fhort of 12 inches †. Now I think it almoft certain, that the quantity of rain that falls yearly in any country, provided it be fufficient for the purpofes of vegetation, muft be very much in proportion to the annual quantity of evaporation in the fame region;

* Difeafes of hot climates.

† And we are not informed that this year was remarkably dry there.

for for a very trifling quantity would any where be enough, if none of it were to paſs off by evaporation; as, on the other hand, ſcarcely any aſſignable quantity would be ſufficient, if the whole of it were ſuddenly exhaled. The dryer the air is, then, in any large extent of country, the more rain is required to ſupport vegetation in its full vigour: ſo that the comparative dryneſs of the atmoſphere, in any two countries, may be pretty fairly inferred from the annual quantity of rain which falls in each of thoſe countries reſpectively, for a courſe of years, provided vegetation be equally vigorous in both.

From the many regiſters which have been publiſhed of the depth of rain, which falls in a great number of places in Europe, and for a long courſe of years, it appears, that the medium quantity of rain in that quarter of the globe ſcarcely equals, but certainly does not exceed, 30 inches from year to year. But in America, viz. at Ipſwich-Hamlet, by the obſervations of the Rev. Mr. Cutler, upon a mean of five years, (the laſt of which, viz. 1787, was rather a dry one) there fell inches 49. 472. And by Dr. Williams's obſervations at Cambridge, there fell, in 1785, inches 47. 616. And by the obſervations of the Rev. Mr.

 French,

French, at Andover, there fell there, on a mean of the feven laft years, inches 51. 2. annually.

The number of fair unclouded days which happen in the courfe of a year, for feveral years together, in any place, muft alfo give fome indication of the drynefs of the atmofphere of that country; for as clouds are formed from the moifture exifting in the air, a freedom from them muft indicate a deficiency of moifture; that is, the air muft be dryer. Now it appears from the *Ephemerides Meteorologicæ Palatinæ*, that the mean number of fair days, by obfervations made in twenty different cities, in different parts of Europe*, amounted to only fixty-three, or fixty-four, and that the fame year, at Cambridge, there were one hundred and feventy-three fuch days. To which I may add, that by my own obfervations at Salem, upon a mean of feven years, we had one hundred and thirty fair days annually.

The number of cloudy days in thefe fame twenty cities was, in 1785, upon a mean, one hundred and thirteen, or one hundred and fourteen; but at Cambridge there were only

* Viz. thofe mentioned in the annexed table.

fixty-

ſixty-nine; and at Salem, upon a mean of ſeven years, about ninety-five days annually.

The number of days, which were partly cloudy, in thoſe ſame cities was one hundred and ſeventy-four, or one hundred and ſeventy-five; at Cambridge but one hundred and twenty-three; and at Salem annually, for ſeven years, one hundred and twenty upon a mean.

The number of rainy days in thoſe cities was upon a mean 122; at Cambridge only 88; and at Salem 95 annually, for ſeven years.

The number of foggy days in thoſe cities was ſixty ſeven; at Cambridge ſixteen; and at Salem, for ſeven years, twenty-one days annually.

As to hygrometrical obſervations, we unluckily have none to compare with the European ones, excepting only thoſe made by the illuſtrious Dr. Franklin, and communicated in a letter to Mr. Nairne, on hygrometers, publiſhed in the 2d vol. of the Tranſactions of the American Philoſophical Society at Philadelphia; by which it appears, that the air of Philadelphia is dryer, not only than that of Great Britain, but alſo than that of Paſſy, in France. This evidence ſeems to be direct.

Our evaporation then is greater than the European; our quantity of rain much greater; we have more clear fair days; we have fewer cloudy days, fewer foggy days, and fewer rainy days*.

Thus, by every method of comparing the two atmofpheres, the American appears to exceed the European in point of drynefs. And although, perhaps, no one of all thefe facts, brought to prove our atmofphere dryer than that of Europe, does, when taken fingly, determine any thing very fatisfactorily, yet, when they are all fairly and candidly laid together, the proof arifing from their joint evidence amounts to a very high degree of probability.

It may now perhaps be thought incumbent upon me to fhow how a greater purity and dry-

* In the fummer feafon, as there are more phlogifticating proceffes going on in Europe, to render the air foul, than in America, fuch as combuftion, refpiration, putrefaction, &c. fo in the latter it is probable, that, at this feafon, the vaft number of trees, in addition to the vegetables, which grow nearer to the earth's furface, in as great plenty as in Europe, muft furnifh a larger proportion of this purer air; fo that in the hot as well as the cold feafons of the year, America muft have the advantage of Europe in this particular.

nefs of the atmofphere fhould occafion greater degrees of cold or heat; or that I fhould point out the procefs of nature in generating heat or cold from drynefs and dephlogiftication *: and many probable reafons drawn from chemiftry, and many very plaufible conjectures, might be adduced, to prove and illuftrate this point. But as it feems generally fuppofed, that all the theories of heat, hitherto propofed, are rather imperfect; or however that may be, as I muft freely confefs myfelf too little acquainted with its nature, to enter upon fuch a difcuffion, I would rather refer to obfervation and experiment.

Now it is, I believe, matter of conftant and univerfal experience, at leaft in this country, and I fuppofe every where in cold countries, that the moft intenfe cold always happens in the pureft, dryeft, and moft dephlogifticated ftate of the atmofphere; or, that we never

* I have ufed the terms *dry*, *pure*, and *dephlogifticated*, as fynonymous, or at leaft have confidered them as qualities accompanying each other in the fame ftate of the atmofphere. But that they are always neceffarily and phyfically connected I do not pretend to affert; that they commonly do accompany each other, I believe to be certain.

have our intenſeſt froſt, but when the air is in this ſtate. That the air is very dry at ſuch times, appears from the ſhrinking of wood, and all vegetable and animal ſubſtances, &c. That it is in a dephlogiſticated ſtate, appears from the rapid conſumption of fuel, and the great tendency to ſcorch obſervable at ſuch times in our ordinary fires; from the increaſed brightneſs and magnitude of the flame of candles and lamps; and from many other circumſtances which might be mentioned. The weather, indeed, is frequently raw cold, as we vulgarly phraſe it, and exceſſively uncomfortable, when the atmoſphere is in a very humid ſtate. The moſt diſagreeably cold weather which we have in winter, happens when the air is in this damp ſtate; but the thermometer at ſuch times is never at or near its loweſt ſtations; perhaps never nearer than 15 degrees, or upwards.

Farther, although the weather is frequently, during ſummer, moſt diſagreeably hot and irkſome to our feelings, when the air is very damp and phlogiſticated, as appears by effects directly oppoſite to thoſe juſt now enumerated, as the conſequences of dephlogiſtication; yet ſo far as my obſervation reaches, the thermometer is never never at its higheſt, at ſuch times, but commonly 6 or 8 degrees below it*.

Whence I think it may fairly be inferred, (whether we are able to account for it philoſophically or not) that dryneſs or dephlogiſtication are, in fact and nature, neceſſary to the production of our intenſeſt cold; and probably of our intenſeſt heat. And if ſo, is it not natural to ſuppoſe, that when the atmoſphere of any country is uſually, both in ſummer and

* It may, perhaps, have the appearance of paradox, to aſcribe two ſuch oppoſite effects, as heat and cold, to the ſame cauſe; but this appearance will, in a good meaſure, vaniſh, if it ſhould be found, as I ſuppoſe it may, that dephlogiſtication produces cold, by its *chemical* effect upon the air: but that it produces heat only *mechanically*, by inducing a more perfectly pellucid ſtate of the atmoſphere, whereby fewer of the ſun's rays are intercepted; and (as dephlogiſticated air is ſpecifically heavier by much than common atmoſpheric air) by occaſioning a greater weight and denſity of the air near the earth's ſurface, whereby the ſun's influence in producing heat is greatly increaſed. Theſe conſiderations may ſerve to ſhow, why cold is ſo much more increaſed by a dephlogiſticated ſtate of the atmoſphere than heat. And it is obſervable, that the difference between two thermometers, one of which is expoſed to the ſun's direct rays, and the other in the ſhade, is always, *cæteris paribus*, much greater in a dephlogiſticated, than in a phlogiſticated ſtate of the atmoſphere.

winter, winter, much dryer, and more dephlogiſticated, that, *cæteris paribus*, it ſhould be hotter in ſummer, and colder in winter there, than in that other?

But, allowing all that has hitherto been advanced upon this ſubject, I would not haſtily conclude, that the ſuperior dryneſs and dephlogiſtication of our atmoſphere is alone ſufficient to account for the whole of our ſuperior heat and cold. There are probably other cauſes, which conſpire with it to produce the ſame effect. I ſhall mention one, which I think of conſiderable moment.

All coaſts which border upon a large ocean, in cold climates, muſt, during the ſeaſon of winter, be warmed by winds which blow from the ocean upon them; plainly for this reaſon, that the waters of the ſea in thoſe latitudes never become ſo cold, by many degrees, as the ſurface of the earth: ſo likewiſe, in thoſe ſame regions, the water of the ſea never becomes, during the ſummer, ſo warm as the earth's ſurface; and therefore, at this ſeaſon, winds blowing from the ſea, upon the land, cool the air.

Now it appears by the *Ephemerides Meteorologicæ Palatinæ*, that the winds which are moſt moſt prevalent in Europe, blow from the Weſt, or at leaſt from that ſemicircle of the horizon; more eſpecially during the ſummer and winter months. Weſterly winds, then, muſt cauſe the air of Europe to be warmer in winter, and colder in ſummer, than thoſe that blow from the oppoſite quarter; becauſe that continent lies eaſtward of the great Atlantic ocean. The directly oppoſite effect takes place in North America from the ſame cauſe; that is to ſay, the winds which prevail moſt with us, particularly in the hot and cold ſeaſons, are likewiſe from the weſtern quarter; for in the vernal and autumnal ſeaſons they are commonly more variable, and blow more frequently from the eaſtward, than in ſummer or winter *. We therefore feel leſs of the warming effects of the ſea air in winter, as well as leſs of its cooling ones in ſummer; becauſe our coaſts lie weſtward of the ocean. Thus the winds which prevail moſt in Europe tend to mitigate both the heat and

* Upon examining a number of American meteorological regiſters for a courſe of ſeveral years, I do not find more than one month, in ſixteen or eighteen, in which eaſterly winds predominate; but I find ſeven or eight, in a year, upon an average, in which they blow almoſt conſtantly from the weſtward.

the cold, to which its geographical ſituation expoſes it; as, on the contrary, the ſame winds increaſe both the cold of winter and heat of ſummer on the American coaſts *.

But upon the ſuppoſition that weſterly winds are moſt prevalent in the middle latitudes all round the globe, which ſeems rather a probable conjecture, if we conſider the facts juſt mentioned; and farther, that the courſe of the trade winds in the torrid zone is continually from the eaſtward, it ought to follow, that the eaſtern coaſt of Aſia, as well as the eaſtern coaſt of America, ſhould be colder than the weſtern coaſt of Europe, or than the weſtern coaſt of America, under the ſame parallel. And that ſuch a difference does really obtain, ſeems to appear from the account given by the writer of Captain Cook's laſt voyage, who informs us that vegetation was in great forwardneſs, in the month of April, at Nootka, or King George's

* Stockholm, in Sweden, lies in lat. 59° 20′ N. and Tobolſki, in Siberia, in 58° 12′; yet it is found by obſervation, that the uſual cold in the latter very much exceeds that of the former. Now, Tobolſki is 50° of long. more eaſterly than Stockholm; of courſe ſo much farther from the Atlantic ocean. Doth not this obſervation confirm the truth of our hypotheſis?

Sound,

Sound, on the weſtern ſide of N. America, in the latitude 49° 36′ N. in the year 1778: whereas the next year, at the bay of Awatſka, in Kamtſchatka, on the eaſtern coaſt of Aſia, in lat. 53° 38′, the ſnows were not gone, nor was there any appearance of vegetation, till the middle of May; which, if to be relied on, as the common courſe of things, is a ſtrong confirmation of the doctrine juſt propoſed *.

But

* It is a common obſervation, among thoſe of our navigators who frequently traverſe the Atlantic, in or near our latitude, that weſterly winds are of all others the moſt uſual; which has occaſioned the ſailors to call the paſſage from the eaſtward *uphill.* And it is obſerved, in Mr. Walter's Account of Anſon's Voyage, that in the Pacific ocean, in the latitude of 30° or 32° North, the winds almoſt conſtantly blow from the weſtward, though in but moderate gales; but that in more northerly latitudes, as 40° or 50°, there are ſteady weſterly winds; the writer therefore ſuppoſes, that the Acapulco ſhip might perform her voyage in much leſs time, if ſhe ſtood farther to the northward, *where weſterly winds conſtantly prevail*, than ſhe does while ſhe purſues her old track. Theſe obſervations are additional proofs of the hypotheſis advanced in this paragraph. Whether weſterly winds prevail in the ſouthern temperate zone, I know not; but if they do, the weſtern coaſt of South America is probably warmer than the eaſtern, in latitudes ſimilar to ours. If the courſe of the winds in our latitudes be generally

But it is more than time to cloſe this paper, already much too long, which I ſhall do, after obſerving, that although I know not whether either or both the cauſes herein ſuggeſted may be judged adequate to the effects which I have aſcribed to them, yet I think we muſt admit the operation of ſome partial or local cauſe (ſuch as greater dephlogiſtication) to account for the greater degree of cold in Europe formerly, than at preſent---as well as of ſome general cauſe (ſuch as the general courſe of winds from the weſtward in the temperate zone) to account for the greater degree of cold on the eaſtern confines of Aſia, than on the weſtern of America.

Be this, however, as it may, I flatter myſelf, that what is here offered may excite ſome pernerally from the weſt, will not this circumſtance alone occaſion the atmoſphere of Europe to be more humid than the American, as the air from the ſea muſt be more charged with the watery vapours, than the land air?

Varenius, in his Geography, page 609, 4th edition, Lond. informs us, that in "the north part of China, though "in a latitude not more northern than Italy, the cold feels "very ſharp, and the great rivers and lakes are frozen."—and page 611, that "in Japan, which extends from 31° to "39° N. they have a cold, ſnowy, wet winter."

ſons

ſons of taſte and leiſure for ſuch inquiries, to attend to the ſubjects here treated, and to examine with freedom the theory here advanced; that ſo, if it ſhall be found agreeable to reaſon and experience, it may be illuſtrated and confirmed; or, if otherwiſe, that it be confuted and exploded.

*Salem, September,* 1788.

### POSTSCRIPT.

Since the Academy did me the honour to read the above paper, I have had the pleaſure of reading in the Philoſophical Tranſactions, Vol. lxxvii. Article xv. an account of ſome very curious experiments made by our countryman, Sir Benjamin Thompſon, at Manheim, in Germany; by which it appears, that eider-down, cotton wool, raw ſilk, &c. yield as much and as pure dephlogiſticated air as the leaves of any kind of vegetables by the ſame proceſs; that is, by expoſing them, when immerſed in water, to the action of the ſun's rays: and therefore, that moſt probably this pure air is derived from the water in which they are thus immerſed, and not from the ſubſtances, whether vegetable, animal, or mineral, which are thus heated. Whence it feems to follow, that it is far from certain that any fuch pure air, or indeed any air at all, is derived from the leaves of plants expofed to the fun, as was fuggefted and feemed to be proved by the experiments of Drs. Prieftley, Ingenhoufz, and others. If this be really the truth of the cafe, and air is not produced from the leaves of vegetables, as in the paper juft now mentioned I have fuppofed it to have been, then all the fubfequent reafoning upon this hypothefis is void of foundation, and muft fall to the ground. But it ought to be noted, that the facts and obfervations contained in the *Eftimate*, which fhow our atmofphere to be really *dryer* than the European, are not at all affected by the failure of this hypothefis, but remain in their full force, though I may have miftaken the caufe when I attributed it to the purity of the air derived from the leaves of vegetables.

Farther, fince writing the paper before mentioned, I have accidentally been informed of a fact, which confirms the idea that our evergreens are, if not the caufe of dephlogifticating the air, yet fomehow the caufe of an increafe of cold. The fact I mean is, *that frofts are commonly obferved to appear much earlier every autumn,*

*tumn, as well as later in the ſpring, in the neighbourhood of pine and other evergreen woods, than in other places, or than in the neighbourhood of other woods which drop their leaves in the winter.* And this I find confirmed by every one I have ſince inquired of, whoſe buſineſs or ſituation leads them to attend to the matter; and I am told, it is a common obſervation, though I confeſs I never heard of it, before I preſented the paper to the Academy.

If this obſervation be well founded, then (whatever may be the fate of Dr. Prieſtley's and Dr. Ingenhouſz's experiments) our pine woods are a ſource of cold which Europe is now in a great meaſure deprived of; as there is no doubt but that trees, of the evergreen as well as of every other kind, are now few, and thinly ſcattered over that continent, compared with what they muſt have been in paſt ages, or than they are in America at preſent.

*Salem, November,* 1790.

*A TABLE of the greateſt HEAT and COLD, and of the Mean of the greateſt Heat and Cold, collected from Obſervations made for a courſe of Years in twenty different Cities in Europe; as exhibited in the* Ephemerides Meteorologicæ Palatinæ: *and at Salem, in North America, for ſeven Years: ſhewing the Exceſs both of Heat and Cold in America, beyond that of Europe in the ſame Latitudes; by* Fahrenheit's *Thermometer.*

| *Names of Places* | Lat. north | Y. of years. | Greateſt Heat by thermom. | Mean of the greateſt heats. | Greateſt Cold by thermom. | Mean of the greateſt Colds. |
|---|---|---|---|---|---|---|
| | ° ′ | | | | | |
| Stockholm | 59:20 | 3 | +87,80 | +83,98 | —16,37 | —10,19 |
| Copenhagen | 55:40 | 4 | +85,10 | +81,77 | + 0,98 | + 2,98 |
| Berlin | 52:32 | 4 | +95, 0 | +89,37 | — 3,55 | — 0,62 |
| Sagan | 51:42 | 5 | +96,35 | +90,86 | —21,32 | —12,23 |
| Erfurt | 51:04 | 5 | +94,10 | +89, 6 | —14, 1 | — 3, 1 |
| Mons | 50:25 | 5 | +95, 0 | +89,15 | — 7,37 | + 1,18 |
| Prague | 50:04 | 4 | +96,35 | +92,07 | —17, 5 | —12,77 |
| Wurtzburg | 49:46 | 5 | +102,4 | +93,87 | —18, 4 | — 4, 0 |
| Manheim | 49:27 | 5 | +93,15 | +89, 6 | — 8,95 | + 1, 2 |
| Ratiſbon | 48:56 | 4 | +96,57 | +79, 7 | —13,45 | — 2,42 |
| Buda | 47:40 | 4 | +92,75 | +90, 7 | — 2, 2 | + 4,26 |
| Peiſſenberg | 47:47 | 5 | +84,35 | +76, 1 | — 8,95 | + 0,28 |
| M. St. Andex | | 5 | +89,60 | +89, 8 | — 7,60 | + 1,13 |
| Tegernſee | 47:37 | 5 | +87,12 | +83, 5 | —12,32 | + 0,73 |
| St. Gotthard | 46:31 | 4 | +66,87 | +62, 3 | —10,75 | — 4,67 |
| Geneva | 46:12 | 3 | +92,75 | +88, 9 | + 1,85 | +10, 2 |
| Rochelle | 46:09 | 4 | +93, 9 | +90, 5 | +10,63 | +16,93 |
| Padua | 45:22 | 5 | +97,25 | +91, 4 | + 7, 7 | +16,93 |
| Marſeilles | 43:17 | 3 | +90, 5 | +89, 6 | +24,13 | +27, 5 |
| Rome | 41:53 | 4 | +86,67 | +85,43 | +31, 1 | +33,46 |
| Mean | | | | +86,41 | | + 3,31 |
| Salem, N. A. | 42:31 | 7 | +106 | +97,02 | —11, 0 | — 2,42 |

XX. *An*

